# Aflatoxins in Uganda: An Encyclopedic Review of the Etiology, Epidemiology, Detection, Quantification, Exposure Assessment, Reduction, and Control

**DOI:** 10.1155/2020/4723612

**Published:** 2020-01-07

**Authors:** Timothy Omara, Winfred Nassazi, Tom Omute, Aburu Awath, Fortunate Laker, Raymond Kalukusu, Bashir Musau, Brenda Victoria Nakabuye, Sarah Kagoya, George Otim, Eddie Adupa

**Affiliations:** ^1^Department of Chemistry and Biochemistry, School of Biological and Physical Sciences, Moi University, Uasin Gishu County, Kesses, P.O. Box 3900-30100, Academic Highway, Eldoret, Kenya; ^2^Department of Quality Control and Quality Assurance, Product Development Directory, AgroWays Uganda Limited, Plot 34-60 Kyabazinga Way, P.O. Box 1924, Jinja, Uganda; ^3^Department of Chemistry, Faculty of Science, Kyambogo University, P.O. Box 1, Kampala, Uganda; ^4^Department of Biochemistry, Faculty of Health Sciences, Lira University, P.O. Box 1035, Lira, Uganda; ^5^Standards Department, Uganda National Bureau of Standards, Plot 2-12 Bypass Link, Bweyogerere Industrial and Business Park, P.O. Box 6329, Kampala, Uganda; ^6^Department of Food Technology and Nutrition, School of Food Technology, Nutrition and Bioengineering, College of Agricultural and Environmental Sciences, Makerere University, P.O. Box 7062, Kampala, Uganda; ^7^Department of Quality Control and Quality Assurance, Leading Distillers Uganda Limited, Plot 3382/83, Buloba, P.O. Box 12369, Kampala, Uganda; ^8^Department of Food Processing Technology, Faculty of Science, Kyambogo University, P.O. Box 1, Kampala, Uganda; ^9^Department of Quality Control and Quality Assurance, Product Development Directory, Sweets and Confectionaries Section, Kakira Sugar Limited, Jinja-Iganga Highway, P.O. Box 121, Jinja, Uganda; ^10^Department of Quality Control and Quality Assurance, Abacus Parenteral Drugs Limited, Block 191, Plot 114, Kinga, Mukono, P.O. Box 31376, Kampala, Uganda

## Abstract

Uganda is an agrarian country where farming employs more than 60% of the population. Aflatoxins remain a scourge in the country, unprecedentedly reducing the nutritional and economic value of agricultural foods. This review was sought to synthetize the country's major findings in relation to the mycotoxins' etiology, epidemiology, detection, quantification, exposure assessment, control, and reduction in different matrices. Electronic results indicate that aflatoxins in Uganda are produced by *Aspergillus flavus* and *A. parasiticus* and have been reported in maize, sorghum, sesame, beans, sunflower, millet, peanuts, and cassava. The causes and proliferation of aflatoxigenic contamination of Ugandan foods have been largely due to poor pre-, peri-, and postharvest activities, poor government legislation, lack of awareness, and low levels of education among farmers, entrepreneurs, and consumers on this plague. Little diet diversity has exacerbated the risk of exposure to aflatoxins in Uganda because most of the staple foods are aflatoxin-prone. On the detection and control, these are still marginal, though some devoted scholars have devised and validated a sensitive portable device for on-site aflatoxin detection in maize and shown that starter cultures used for making some cereal-based beverages have the potential to bind aflatoxins. More efforts should be geared towards awareness creation and vaccination against hepatitis B and hepatitis A to reduce the risk of development of liver cancer among the populace.

## 1. Introduction

### 1.1. Brief Historical Perspective

Aflatoxin (AF) is a portmanteau combining “a” for the *Aspergillus* genus, “fla” for the species *flavus*, and *toxin* for poison [1–3]. The discovery of aflatoxins traces back to 1960 in which a severe outbreak of Turkey “X” disease was recorded in England with more than 100,000 turkeys, 20,000 ducklings, pheasants, chicks, and partridge poults reported to have died from the calamitous incident [4]. The cause was reported to be due to a series of fluorescent compounds in a peanut meal imported from South America (Brazil) that was served to the poults [5]. Later, the disease syndrome was reported in domesticated animals outside Great Britain. The causative mold, *Aspergillus flavus,* was finally isolated from a meal later related to a hepatic problem in ducklings in Uganda [6]. The early history of the Turkey “X” disease outbreak in Great Britain was described in sufficient details by Blount [4, 7], and the toxicity recorded in various animal species was recapitulated by Allcroft [8].

### 1.2. Structure and Properties of Aflatoxins

Aflatoxins are highly oxygenated polysubstituted coumarins with structures that differ only very slightly. At least 18 different types of AFs have been chemically characterized (Table 1), with the six major ones being aflatoxin B_1_ (AFB_1_), aflatoxin B_2_ (AFB_2_), aflatoxin G_1_ (AFG_1_), aflatoxin G_2_ (AFG_2_) [11], aflatoxin M_1_ (AFM_1_), and aflatoxin M_2_ (AFM2) (Table 1). The B-aflatoxins, typically pentanone derivatives, exhibit strong blue fluorescence under ultraviolet light while the G-series (six-membered lactones) fluoresce yellow-green on thin-layer chromatography plates, thus the B and G designations [12, 13]. AFB_2_ and AFG_2_ are dihydroxy derivatives of AFB_1_ and AFG_1_, and the other AFs are not usually reported in the absence of AFB_1_ [14]. The M series are toxic metabolic derivatives of the B series that exhibit blue-violet fluorescence and have been reported in the milk of animals fed with AF-contaminated feed [15, 16], hence the designation M [9, 12, 17–19]. The subscripts 1 and 2 in AF nomenclature are designations for major and minor, respectively. The minor AFs have received description as mammalian biotransformation products of the major metabolites [20].

Aflatoxins are produced mainly by *Aspergillus flavus*, *A*. *parasiticus*, *A*. *nomius*, and *A*. *tamarii* [21–24] which are universally soilborne fungi responsible for decomposition of plant materials. About 20 *Aspergillus* species have been reported to produce AFs [25], though the exploration of more novel and potential aflatoxigenic fungi continues [26–31]. Most species produce B-type AFs via the polyketide pathway as difuranocoumarin derivatives although species related to *A*. *parasiticus*, *A*. *nomius*, *A*. *toxicarius*, *A*. *bombycis*, *A*. *parvisclerotigenus*, *A*. *minisclerotigenes*, and *A*. *arachidicola* are able to additionally produce G-type aflatoxins [32]. AFM_1_, AFM_2_, AFB_2A_, and AFG_2A_ have been isolated from cultures of *A. flavus* and *A. parasiticus* while AFGM_1_, parasiticol, and aflatoxicol are solely produced by *A. flavus* [16].

Chemically, AFs are unique highly substituted coumarins containing a fused dihydrofurofuran moiety [33]. The B-series are characterized by fusion of a cyclopentenone ring to the lactone ring of the coumarin moiety whereas the G-series contain a fused lactone ring [34]. AFB_1_ and AFG_1_ possess an unsaturated bond at the 8, 9 position on the terminal furan ring, and some studies illustrated that oxiranation at this chemical position is pivotal for their toxicological potency. AFB_2_ and AFG_2_ are comparatively less toxic, unless they are first oxidized to AFB_1_ and AFG_1_* in vivo* [33]. AFs are soluble in polar protic solvents [15].

### 1.3. Toxicological Properties of Aflatoxins

In kingdom Animalia, AFs are reported to be multiplicatively carcinogenic, genotoxic, tremorgenic, haemorrhagic, dermatitic, mutagenic, teratogenic, and immunosuppressive [11]. They display potency of toxicity, carcinogenicity, and mutagenicity in the order: AFB_1_ > AFM_1_ > AFG_1_ > AFB_2_ > AFM_2_ >AFG_2_ as exemplified by their lethal dose that causes the death of 50% of subjects (LD_50_ values) being 0.1–50 mg/kg body weight for most animal species and <1.0 mg/kg body weight for susceptible species [35–37] (Table 2). The order also reflects the role played by the epoxidation of the 8,9-double bond and the greater potency associated with the cyclopentenone ring of the B-series. Trial tests on animal species and mammalian cells have unveiled toxicities of AFG_1_, AFB_2_, and AFG_2_ as approximately 50%, 20%, and 10% that of AFB_1_ [38]. Susceptibility though varies with breed, species, age, dose, length of exposure, and nutritional status of the exposed animals (Table 2).

AFB_1_ is listed as a human class 1 carcinogen [40, 41] and the most potent carcinogen known [42, 43] that may play a part in the etiology of human liver cancer. This is due to its demonstrated ability to bind to nucleic acids (DNA and RNA) and proteins [40, 44, 45]. The carcinogenicity of AFs has been shown to operate by a genotoxic mechanism involving metabolic activation to a genotoxic epoxide metabolite, formation of DNA adducts, and modification of the TP53 gene which involves the transversion of guanosine to thymine [43]. AFs interact with basic metabolic pathways of the cell, disrupting key enzyme processes including carbohydrate and lipid metabolism as well as protein synthesis. It is unfortunately reported that where AFs are detected in foods, AFB_1_ usually exceeds half the total amount present, explaining the reason why compliance limits for AFs include AFB_1_ and several analytical methods have been developed and validated to quantify its concentration in foods [46]. Aflatoxin M_1_, like AFB_1_, is a classified group 2B probable human carcinogen [47].

Human exposure to AFs has documented deleterious health effects including acute aflatoxicosis and chronic exposure leading to liver cancer with 8.19 cases reported per 100,000 inhabitants in Africa annually [40]. About 3,700 of these cancer cases are from Uganda [41]. In fact, the risk of developing liver cancer is reported to be high (50% more) in cases where the individuals are carriers of hepatitis B and hepatitis C surface antigens [42]. In addition, AFs impair protein synthesis and induce coagulation, weight gain, and immunogenesis [39].

Food-borne AFs have been implicated for inducing infantile stunting [43, 44] probably by interfering with protein synthesis and the activity of micronutrients (vitamins: A, B_12_, C, D, and E, zinc, selenium, iron, and calcium) [16]. Diminished feeding and weight loss have been reported in domesticated animals fed on AF-contaminated feed [43], ensued by death. AFs also cause lower milk and egg production as well as immune suppression due to the reaction of AF with T cells (perforin, perforin-expressing, and granzyme A-expressing CD8^+^ T cells) [45] and a decrease in vitamin K activities [39].

All these have economic impacts, extensible to the national economy, estimated at 128 billion annually for Uganda [46]. In 2013, more than 600,000 tons of maize worth Uganda shillings 10 billion destined for export to neighbouring Kenya was rejected because they had AFs above regulatory limits [47].

## 2. Etiology of Aflatoxins in Uganda and the Commodities Contaminated

### 2.1. Etiology

In Uganda, AFs are produced predominantly by *A*. *flavus* and *A. parasiticus* [48]. *A*. *flavus* is ubiquitous and is reported to produce AFB_1_ and AFB_2_ along with other mycotoxins: cyclopiazonic, kojic, and aspergillic acids [32]. *A. parasiticus* produces AFB_1_, AFB_2_, AFG_1_, and AFG_2_ accompanied by mycotoxic kojic and aspergillic acids [32, 49, 50].

The climatic conditions in Uganda such as heavy rains, sudden droughts, high humidity, average temperature of 25°C, occasional floods as well as poor pre-, peri, and postharvest handling of foods by farmers and traders in the food value chain have been implicated for the proliferation of AFs in Ugandan foods [48].

Biophysical factors such as soil (substrate composition), crop species (host-plant susceptibility and genotype), and fungal populations (strain specificity and variation, instability of toxigenic properties) as well as levels of education, awareness, and gender are another probable set of factors contributing to AF contamination and prevalence in agricultural foods in Uganda as reported elsewhere [51, 52]. Other factors that may influence AF production include water activity, pH, surrounding concentration of oxygen and carbon dioxide, microbial competition, mold lineage, plant stress, and use of fungicides or fertilizers.

*A. flavus* and *A. parasiticus* are semithermophilic and semixerophytic, thriving favorably between 12°C and 48°C and at lower water potentials [53]. Optimum growth occurs between 25°C and 42°C and low water activity associated with droughts as in Uganda. These factors contribute to the epidemiology of the two *Aspergillus* fungi. Despite the optimum temperature for AF biosynthesis reported to be between 28°C and 35°C, some studies indicate higher temperatures inhibit AF biosynthesis [54, 55]. Thus, the conditions in Uganda favor *A*. *flavus* and *A*. *parasiticus* growth along with their aflatoxigenic contamination of foods.

In Uganda, AFB_1_ is the most studied [56] while AFM_1_ has received little attention [57]. Thus, most studies reported AFB_1_ levels or did not distinguish between the different types [58–63]. Others, such as the validation survey of Wacoo et al. [64], Muzoora et al. [65], Baluka et al. [66], and Wacoo et al. [67], differentiated the AFs. By and large, the lack of this depth in most studies can be tailored to the overall priority of simply analyzing the safety of foods and/or individuals. More so, there was a limited facility to handle AF analysis as well as lack of funds to procure the analytical grade reagents [48]. Despite the documented differences in toxicity, all AFs are harmful and should be detected, quantified, and rigorously controlled. Further, there is a dire need for comprehensive and coherent data on potential mycotoxins [68].

### 2.2. Commodities Contaminated

Aflatoxigenic contamination in Uganda has been reported in maize (*Zea mays* L.) [59, 62, 63, 69], sorghum (*Sorghum bicolor* L.), finger millet (*Eleusine coracana*) and their local products [58], peanuts (*Arachis hypogaea* L.) [57, 65, 66, 70], cassava (*Manihot esculenta*) [71], rice (*Oryza sativa*) [72], sunflower (*Helianthus annuus*), sesame (*Sesamum indicum* L.) [63], animal feeds [73], and a bovine milk-based product [57]. AFs have also been detected in human sera [60, 61, 74]. Virtually all grains, spices, and other oil seeds cannot be exempted [47].

#### 2.2.1. Peanuts (*Arachis hypogaea* L.)

Peanuts (groundnuts) are the only cheap source of plant proteins, second in importance to beans and majorly cultivated in Eastern and Northern Uganda but consumed countrywide [75]. It is consumed as seeds, raw, roasted, blanched, peanut butter, or mixed with traditional dishes as a sauce or as *ebinyewa* (paste or flour) [76].

Lopez and Crawford [70] reported on the AF content of peanuts sold for human consumption in Uganda. On average, 15% of the samples had more than 1.0 *μ*g/kg of AFB_1_ while 2.5% contained more than 10 *μ*g/kg of AFB_1_. The contamination levels were at peak at the end of the rainy season prior to the new harvest season. Further, Korobkin and Williams [77] reported the need for AF analysis of peanuts consumed by the community of West Nile as investigation of primary liver carcinoma and groundnut growing regions of Arua showed some correlation between cancer cases reported in the tumor registry of Kuluva Hospital (between 1951 and 1965) to the distribution of the peanut growing areas.

Total AFs were reported in 80% of peanut and peanut paste samples traded in metropolitan Kampala with 40% of these having AF content exceeding FDA/WHO compliance limit of 20 *μ*g/kg by Osuret et al. [78]. Unprecedented AF levels (940 *μ*g/kg and 720 *μ*g/kg) were reported in peanut paste and peanut seeds, respectively.

The aforeacknowledged studies never correlated the AF contaminations with their causes. Subsequently, Kaaya et al. [79] reported in a correlative study that at farm level in villages, up to 60% of peanuts had detectable AFs (Table 3). Further, low levels of awareness, poor storage practices, and poor processing practices (drying, sorting, and milling) were implicated for the heightened AF levels registered, stressing that aflatoxigenic contamination commences right from farms. Comparative analysis of market peanuts unveiled significantly higher total AF contents in retailed samples than those wholesaled.

In a similar concerted study [57], up to 100% of peanut flour samples used in Southwestern Uganda culinary recipes were reported positive for total AFs with a mean of 11.5 ± 0.43 *μ*g/kg (Table 4). Lack of awareness and knowledge of AF contamination control were reported to be the probable reasons for the high AF levels recorded.

Muzoora et al. [65] screened 120 peanut samples sourced from Ugandan districts of Kampala, Mubende, Gulu, Pader, Mbarara, Masindi, and Kaberamaido for AFs followed by competitive enzyme-linked immunosorbent assay (ELISA) quantification. Their report indicated that 72% of the samples were AF-positive with 26% having AFB_1_, AFB_2_, AFG_1_, and AFG_2_ whereas AFB_1_ and AFG_1_ containing samples constituted 74% of the total samples. More urban samples (67.1%) were AF-positive than rural samples (47.6%). ELISA gave 81% AF-positive samples, with milled groundnuts registering higher total AF (range: 0.31 to 1,1732 *μ*g/kg; mean: 1,277.5 ± 382.2 *μ*g/kg) compared to whole groundnut seeds (range: 1.6 to 516 *μ*g/kg; mean: 84.7 ± 43.8 *μ*g/kg). Up to 52% of the samples in the study registered total AF contents greater than the FDA/WHO maximum compliance limit of 20 *μ*g/kg for total AF in peanuts. There were typically no significant differences reported in the AF content of peanuts from the different regions. The study implicated milling of fungal contaminated peanuts by traders to shield evidence of spoilage from consumers and the skewed distribution of AF in the studied matrices for the reported relative differences in AF levels of milled and whole peanuts.

Partnership for Aflatoxin Control in Africa (PACA) report [80] indicates that peanuts in Uganda are mycotoxicologically unfit for human consumption. Kioga plains (Iganga and Soroti districts) in a survey had 20% of the peanuts with AF levels above 10 *μ*g/kg while Tororo had 10% of the samples above the regulatory limit of 10 *μ*g/kg. In addition, other agroecological zones had 10% of peanut samples with AF contamination in levels above 10 *μ*g/kg except for Northeastern which had none of the samples with detectable AFs. The report is substantiated by investigations of Baluka et al. [66] which reported that 34% of 55 peanut samples analyzed in a study contained AFs in concentrations greater than the East African and FDA/WHO compliance limits for AFs in peanuts.

From the foregoing reports, it can be noted that very high concentrations of AFs have been reported in peanuts in Uganda. This could be because as the pods grow in the soil, various aflatoxigenic fungi contaminate the shells, testa, and seeds. Worse still, mechanical damage during harvest, drying, and storage further increases the chances of fungal contamination and mycotoxin production. This is substantiated by a study which revealed that grains and oilseeds from maize, sorghum, and sunflower produced in above the ground reproductive structures had relatively lower AF contamination compared to those produced in geocarpic structures of groundnut and Bambara nut [81].

#### 2.2.2. Cereals (Maize, Millet, Sorghum, and Rice) and Cereal-Based Products

The occurrence of mycotoxins and associated aflatoxigenic *A. flavus*/*A. parasiticus* in staple Ugandan foods and their derivative poultry feeds were evaluated by Sebunya and Yourtee [73]. The fifty-four (54) samples of maize, peanuts, soybean, and poultry feed samples taken and precultured on *A. flavus*/*parasiticus* selective agar (AFPA) were analyzed for their fungal content on a coconut agar medium under ultraviolet light with a subsequent confirmatory scrutinization for AF production in a pure culture. Twenty-five (25) of the samples were analyzed for AFB_1_, AFG_1_, zearalenone, sterigmatocystin, ochratoxin A, citrinin, vomitoxin, and diacetoxyscirpenol. *A. flavus/parasiticus* were reported in 77% of maize and peanuts (36% human food; 83.3% animal feed) and 66.6% in poultry feed. No fungus was detected in soybeans whereas two (8%) of the 25 mycotoxin-scrutinized samples had 20.0 *μ*g/kg of AFB_1_ (4 times the statutory limit of 5.0 *μ*g/kg for AFB_1_ in Ugandan foods).

Five baby food products locally produced in Uganda were bought from different shops and supermarkets at the stage of consumption and investigated for contamination by different toxigenic fungi and AFs by Ismail et al. [80]. These foods, each with one or more cereal flour as an ingredient, were cultured using the dilution plate method and three selective isolation media (pentachloronitrobenzene rose Bengal yeast extract sucrose agar (PRYES), peptone-pentachloronitrobenzene agar (peptone-PCNB), and AFPA) and enumerated. PRYES plates revealed a high level of contamination of the foods by *Penicillium*, with three species being nephrotoxigenic (*P*. *viridicatum*, *P*. *verrucosum*, and *P*. *citrinum*). On the one hand, nine species of *Fusarium* were recovered in high frequencies and counts on peptone-PCNB. Of these, *F. verticillioides* followed by *F*. *solani* were the most prevalent while *F. proliferatum* and *F*. *tricinctum* had more propagules. In addition, aflatoxigenic *Aspergilli* were isolated on AFPA from the majority of samples of all the products investigated. *A. flavus*, *A. niger*, *Cladosporium*, and yeasts were prevalent. Regarding total AFs, all samples analyzed were contaminated, though the levels detected were below or in the current tolerance level of 10 *μ*g/kg and 20 *μ*g/kg accepted in foodstuffs by Ugandan standards and WHO/FDA, respectively. The contaminated foods constitute a health hazard to babies as they have a more restricted diet and generally consume more food on a body weight basis than adults. They concluded that the foods must be examined regularly to assess their quality.

Lee et al. [69] reported that 11% of 55 maize samples collected in a survey were contaminated with AF in the range of 12.7–123.5 mg/kg, 9% of which exceeded the maximum regulatory limit. PACA [78] reported that sorghum from the different agroecological zones represented by Lira, Gulu, Amuria, Soroti, and Tororo districts of Uganda recorded between 90 and 100% of the samples positive for AFs, with total AFs ranging from 4.0 to 265.5 *μ*g/kg (mean from 11.5 to 170.1 *μ*g/kg). Between 85% and 100% of the samples registered total AF greater than 4 *μ*g/kg, while between 70% and 100% of the samples had AF greater than 10 *μ*g/kg. Between 65% and 100% of the samples had AF content greater than 20 *μ*g/kg. Kitya et al. [57] further reported that millet and sorghum from Southwestern Uganda had mean total AF contents of 14.0 ± 1.22 *μ*g/kg and 15.2 ± 0.2 *μ*g/kg, respectively (Table 4). A regional report cited in [82] indicates that maize in Uganda is the least contaminated in the East African Community (Table 5).

The moisture and total aflatoxin content of 27 samples of fresh harvested maize from Mubende, Ibanda, Jinja, Mayuge, Buikwe, Hoima, Mpigi, Masindi, and Bugiri districts of Uganda representing the agroecological zones: Lake Victoria crescent, Western Highlands, South East, and Lake Albert crescent were determined by Omara [65]. The moisture content ranged from 12.9% to 18.8% (mean: 13.9 ± 0.35% to 17.2 ± 1.55%) with the highest moisture recorded in maize from Ibanda. The highest mean AF content of 11.0 ± 3.01 *μ*g/kg was recorded in maize from Hoima while the lowest AF content of 3.8 ± 1.30 *μ*g/kg was reported in maize from Mpigi. All the samples had detectable AFs, but none had AF content greater than 20 *μ*g/kg. The lower levels of aflatoxin recorded in this study were attributed to the fact that the maize had not undergone postharvest handling practices which are reported to increase AF content in maize [58]. The study concluded that maize in Uganda is precontaminated by AFs prior to harvest and recommended that farmers should plant maize varieties with established maturity periods to ensure timely harvesting.

#### 2.2.3. Cassava (*Manihot esculenta* L.)

Cassava is one of the most important staple foods in Uganda grown majorly in Northern Uganda and Eastern Uganda [81]. The dynamics in cyanogen levels during the processing, the associated microflora, proteinaceous content, amino acid patterns, and mycotoxin contamination of cassava products processed traditionally by the Alur people of West Nile were investigated by Essers et al. [83]. Cassava tuber processing was monitored at six rural households and replicated in an analytical laboratory setting, comparing it to sun-drying. Cassava flours from the rural households were analyzed for residual cyanogens, mutagenicity, cytotoxicity, and AFs. No AFs were detected in the samples.

Data available in open literature have reported AF contamination of cassava in Uganda at an average content of 0.5 *μ*g/kg (Table 5). Osuret et al. [78] found 20% (1/5) samples of cassava sold in metropolitan Kampala to be aflatoxigenically contaminated in levels above the WHO/US EPA compliance limit of 20 *μ*g/kg. In a similar concerted investigation, Kitya et al. [57] bewrayed that cassava chips in Southwestern Uganda are mycotoxicologically contaminated with a mean total AF content of 16.0 ± 1.66 *μ*g/kg. Kaaya and Eboku [71] reported *Rhizopus* (66.7%), *Mucor* (37%), *Penicillium* (22.2%), *Aspergillus* (20.4%), and *Fusarium* species (5.6%) as the fungi contaminating dry cassava chips in Eastern Uganda with up to 30% of the samples registered positive for AF (mean total AF content was 0.51 *μ*g/kg; AF range was 0.0 to 4.5 *μ*g/kg). *A*. *flavus* regrettably was reported in 18.5% of the analyzed samples.

#### 2.2.4. Animal Products

Most of AFB_1_ and AFB_2_ ingested by mammals are eliminated through urine and faeces. A fraction of this is biotransformed in the liver and excreted in milk and urine as AFM_1_ and AFM_2_, respectively. AFM_1_ is detectable in milk 12–24 hours after the first AFB_1_ ingestion, reaching a high level after a few days. Thus, dietary exposure to AFs through consumption of milk from lactating animals fed on AF-contaminated feeds in Uganda is as high as microbial contamination of milk reported in Metropolitan Kampala [84]. In Western Uganda, Kitya et al. [57] reported that a bovine milk-based ghee sauce (*Eshabwe*) had a mean total AF content of 18.6 ± 2.4 *μ*g/kg which was the highest of all the matrices tested for AFs in the Ankole districts of Mbarara, Ntungamo, Rukungiri, Kasese, and Kabale (Table 4). *Eshabwe* is a traditional Ankole delicacy prepared from unprocessed ghee, rock salt, boiled cold water, and salt and is commonly prepared for special ceremonies as a condiment [85]. Given the fact that this sauce is almost prepared by every Ankole family, the study indicated that the high incidences of hepatocellular carcinoma could be correlated to the consumption of such aflatoxin-contaminated foods resulting from the traditional food processing techniques [57].

Upon ingestion of AFB_1_, cytochrome P450 enzymes (CYP) (including CYP1A2, CYP3A4, and CYP2A6) in the liver and other tissues convert AFB_1_ to epoxides (AFB_1_-8,9-exo-epoxide and AFB_1_-8,9-endo-epoxide) and to AFM_1_, AFP_1_, AFQ_1_, and its reduced form aflatoxicol. Of the epoxides, the AFB_1_-8,9-exo-epoxide can form covalent bonds with DNA and serum albumin resulting in AFB_1_-N7-guanine and lysine adducts, respectively. Like AFB_1_, AFM_1_ can be activated to form AFM_1_-8,9-epoxide that binds to DNA resulting in AFM_1_-N7-guanine adducts. Guanine and lysine adducts have been noted to appear in urine. The metabolites AFP_1_, AFQ_1_, and aflatoxicol are thought to be inactive and are excreted as such in urine, or in the form of glucuronyl conjugates from bile in faeces [86].

In Uganda, there is no report on the aflatoxin content of other products of animal origin such as meat and blood.

### 2.3. Co-Occurrence of Aflatoxins with Other Mycotoxins in Ugandan Foods

Several mycotoxins can occur simultaneously in matrices [87]. The statutory and regional regulations in place for food and feed products are based entirely on AFs, failing to take into consideration possible combined toxic effects of different mycotoxins. Some studies in Uganda have reported the co-occurrence of AFs with some mycotoxins. In an investigation by Sebunya and Yourtee [72] on 25 samples of foods analyzed for AFB_1_, AFG_1_, zearalenone, sterigmatocystin, ochratoxin A, citrinin, vomitoxin and diacetoxyscirpenol, zearalenone, and vomitoxin were detected in 3 and 2 maize samples, respectively.

Following a WHO meeting on nodding syndrome in Kampala (Uganda) in 2012, it was recommended that fungal contamination of foods should be investigated as a possible cause of the disease. Echodu et al. [63] assessed the relationship between consumption of mycotoxin-contaminated foods (sorghum, millet, sunflower, groundnut, sesame, and maize) and the development of nodding syndrome in the affected Northern districts of Lamwo and Kitgum. Very high levels of total AFs and ochratoxins in millet, sorghum, maize, and groundnuts in both households with and without children with nodding syndrome were registered. No significant association between concentrations of the mycotoxins and the presence of children with nodding syndrome in households was noted. Sorghum in this study had the highest total AF ranging from 0.00 to 68.2 *μ*g/kg while the lowest AF was recorded in sesame (maximum AF of 4.5 *μ*g/kg). In this study, the highest ochratoxin and vomitoxin/deoxynivalenol contents were 7.647 *μ*g/kg and 2.606 *μ*g/kg reported in sorghum and maize from Lamit Tumangu village, Kitgum district, respectively.

Baluka et al. [66] compared mycotoxins and selected trace metal content of peanuts sold in selected markets in Kampala, Uganda, to those traditionally prepared. Commercially processed peanut samples (*n* = 33) were purchased from St. Balikuddembe, Nakawa, Kalerwe, and Bukoto markets of Metropolitan Kampala whereas control samples (*n* = 5) were unground peanuts procured from the markets and processed using traditional methods or by metal grinding. Aflatoxins: B_1_, B_2_, G_1_, G_2,_ fumonisins, deoxynivalenol, nivalenol, ochratoxin A, T2 toxin, zearalenone, zearalenol, and heavy metals: arsenic, boron, barium, cadmium, chromium, copper, mercury, magnesium, nickel, lead, and zinc were analyzed. AF, particularly AFB_1_, was reported as the predominant mycotoxin in the samples. There were significantly higher concentrations of AFs in market-processed than in home-processed samples. AF concentrations were in the range of 0–540 *μ*g/kg for AFB_1_, 0–141 *μ*g/kg for AFB_2_, 0–213 *μ*g/kg for AFG_1_, 0–36 *μ*g/kg for AFG_2_, and 0–849 *μ*g/kg for total AFs. Cadmium and lead content of the samples were below the method limit of detection of 0.25 ppm though one sample (2.6%) had arsenic above the FDA maximum concentration of 1.4 ppm. The concentrations of chromium and mercury in 100% of the samples were below the FDA limit of 1 and 0.5 ppm, respectively. Roasting and duration of grinding had no appreciable effect on AFs and metalliferous content of the samples. The study recommended the need for food-borne toxicant monitoring of foods traded for human consumption in Ugandan markets [66].

### 2.4. Geographical Distribution of Aflatoxins in Uganda

Brazil was the first hotspot of AFs recorded [88] before subsequent reports cited Uganda, Kenya, Senegal, Mozambique, Swaziland, Nigeria, China, Thailand, and the Philippines [89]. Sherck-Hanssen [90] reported in 1970 a case report that implicated the death of a Ugandan to be linked with ingestion of aflatoxin-contaminated cassava. The 15-year-old boy was admitted to Mulago Hospital, Kampala, on June 4, 1967, with abdominal pains and swelling of the legs for a couple of days. The pulse rate was declared normal. Probing clinical analyses reported that he was having heart failure. Upon administration of digitoxin and mersalyl sodium, the boy passed away two days after admission. An autopsy recorded edema and congestion of the lungs with diffuse necrosis of the liver. Histology revealed centrilobular necrosis, and subsequent aflatoxigenic investigation of a sample of the cassava eaten by the boy with his sister and brother (who also became ill but survived) indicated the cassava had 1,700 *µ*g/kg of aflatoxin B_1_ which is markedly lethal if ingested for over three weeks when compared with the acute toxicity dose of 220 *µ*g/kg AFB_1_ in African monkeys [91].

Uganda is divided into ten agroecological zones: Southern highlands, Southern dry lands, Lake Victoria crescent, Eastern, Mid-Northern, Lake Albert crescent, West Nile, Western highlands, South East, and Karamoja drylands [92]. AFs tend to be recorded at nearly equal concentrations in food samples from the different zones. This can be attributed to the similarity in the agronomic, pre-, peri-, and postharvest handling practices and the interregional marketing of foods in Uganda [80].

In one of the pioneering surveys, the AF content of 480 foods stored for consumption between harvests in Uganda between September 1966 and June 1967 was evaluated by Alpert et al. [93]. Up to 29.6% of these had detectable AF with 3.7% of the samples recording >1.0 *μ*g/kg AF content. Beans had the highest AF content (72%), while the prevalence of aflatoxins in maize, peanuts, and cassava was reported at 45%, 18%, and 12%, respectively. Rice in this study had no detectable aflatoxins. The high prevalence of aflatoxigenic contamination reportedly correlated with provinces with a high recorded hepatoma incidence or moldy food consumption (Table 6). This led to the postulation that AF exposure may be a contributing factor for the elevated levels of hepatoma in Uganda [89]. In the same study, the local cancer registry in the regions where samples were drawn was checked for the period 1964 to 1966. The study indicated that the Karamoja region had the highest hepatoma frequency of 6.8 cases per 1,000 people per annum with a frequency of AF contamination at 44% (Table 7). Overall, hepatoma occurred at an average rate of 1.0 to 2.7 cases per 1,000 people per year [93].

No study has reported in the open literature on the AF content of beer consumed by Ugandans, yet it is among the most consumed foods that perhaps use all the major cereals: maize, sorghum, and barley as well as cassava. Beers are practically products of mixed-culture fermentations, a process that continues up to consumption time. Thus, brewing is an ideal route for exposure to AFs as it offers favorable conditions for aflatoxigenic fungal growth [90] and creates an avenue for use of contaminated grains as the final consumers will not be able to physically detect as reported for peanut paste [65].

## 3. Capacity for Detection and Quantification

Specific, sensitive, and simple analytical methods for detection and quantification of AFs are prerequisites for their accurate detection and quantization given their presence in very meagre concentrations and their skewed nature of distribution in matrices [94]. The accuracy, precision, reproducibility, and repetitiveness of analytical techniques for detection and quantification of the AF content of a commodity are largely influenced by the way each step in the analytical process from sampling to extraction, cleanup, and quantification is perfected. One of the biggest challenges is that it is often hard to obtain representative samples for AF analysis for bulk lots of commodities. This is in part due to the fact that the aflatoxigenic molds do not grow uniformly in the matrices, giving a skewed distribution [94].

### 3.1. Methods of Detection and Quantification Employed by AF Investigations in Uganda

The methods for the detection of AFs in agricultural foods have been reviewed in sufficient details by some Ugandan authors [15]. This also explains, in part, the fact that most AF investigations in Uganda following this review such as that of Muzoora et al. [65], Echodu et al. [63], Wacoo et al. [67], and Byakika et al. [58] employed selective and highly sensitive methods.  Table 8 summarizes some of the methods employed by aflatoxigenic investigations in Uganda.

Generally, aflatoxigenic analysis of samples employed laboratory-based high-performance liquid chromatography (HPLC), thin-layer chromatography (TLC), enzyme-linked immunosorbent assays (ELISA), fluorescence spectrophotometry (FS), and liquid chromatography-tandem mass spectrometry (LC-MS/MS) which are expensive, labour-intensive, and time-consuming [15]. Unlike reported before [48], Uganda has developed some appreciable capacity to detect and quantify specific AFs with laboratories at Makerere University, Chemiphar Uganda Limited, Uganda National Bureau of Standards, Uganda Industrial Research Institute, and Directorate of Government Analytical laboratory. Unfortunately, all these laboratories are in the country's capital (Kampala) making them inaccessible to other regions. At industrial level, agroprocessing companies are monitoring total AFs in maize using single-step lateral flow immunoassays utilizing Reveal Q+ test strips that are developed and read on AccuSan Gold readers [46, 62].

Due to limited access to the aforelisted laboratory-based analytical methods, a rapid on-site AF portable immunosensor based on a glass-electroless-plated silver/cysteine platform for detection of total AF was constructed at Uganda Industrial Research Institute, plot 42A, Mukabya Road, Nakawa, Kampala, Uganda, by Wacoo and his teammates [97]. This electrochemical immunosensor device was subsequently validated in a penultimate study [64] which assessed the AF content of 60 maize flour samples in six principal markets and 72 samples from selected households in Metropolitan Kampala. The immunosensor was validated with a linear range of 0.7 ± 0.1 to 11.0 ± 0.3 *µ*g/kg and limit of detection of 0.7 ± 0.0 *µ*g/kg. Maize flours from the scrutinized markets of Usafi, Nakawa, St. Balikuddembe (also called Owino), Nakasero, Kireka, and Kalerwe had a mean total AF of 7.6 ± 2.3 *µ*g/kg with approximately 20% of the samples having higher than 10 *µ*g/kg statutory AF limit while 45% of household samples had total AF above compliance limit. The AF results from the immunosensor reportedly correlated with HPLC and ELISA results with correlation coefficients of 0.94 and 0.98, respectively [64].

Bright greenish-yellow fluorescence (BGYF) or the black light test, which can locate lots presumed to be contaminated with AF, has not been reported in Uganda. This is a simple test for AF in maize where kernels are viewed under an ultraviolet lamp at 365 nm for characteristic bright greenish-yellow fluorescence. This indicates a possible presence of aflatoxigenic fungi or the mycotoxin itself [98]. Regulatory bodies in Uganda should develop the capacity to perform this simple detection test for surveillance surveys.

### 3.2. Exposure Assessment

Humans are exposed to AFs through oral ingestion of contaminated plant products (such as peanuts) primarily as AFB_1_ or animal products such as meat and milk from animals previously fed on AF-contaminated feed (in the form of AFM_1_) [16]. Farmers and other agricultural workers may also get exposed by inhaling dust generated during the handling and processing of contaminated crops and feeds.

Analytical detection and quantification of AFs in foods do not give the exact exposure levels as the quantities detected in raw foods are not necessarily equivalent to that ingested. Losses are possible, and therefore, epidemiological biomarkers on dietary exposure have been employed to assess the level of exposure. Biomarkers are more precise for assessing the degree of exposure to AFs, as they are nonsubjective and can determine the internal and biologically effective doses. Aflatoxin biomarkers in use currently include the AF-N7-guanine adducts excreted in urine (reflect the previous day's exposure), AFM_1_ (primarily in breast milk, and reflects exposure over the previous 24 hours), and the aflatoxin-albumin adduct (AF-alb) in plasma or serum with half-life of about 2 months which allows assessment of chronic and routine exposure to AFs [99]. Albumin, the only serum protein that binds AFB_1_, forms a high level of adducts [100], while haemoglobin binds AFB_1_ in a very low yield [101]. Albumin extracted from human blood and urine avails a measure of the biologically effective dose of ingested AFB_1_. AFB_1_ and AFG_1_ can be bound by albumin and are metabolized to 8, 9-epoxide [102]. The AF-alb adduct levels are considered as AFB_1_ amount ingested as AFG_1_ is less prevalent in foods [38]. Thus, the AF-alb biomarker is the more commonly employed as it can be easily detected by ELISA (with results in pg AF-alb/mg albumin or in pg AF-Lys equivalent/mg alb) [103]. Quantification of AFB_1_-Lys in proteolytic digests of serum with HPLC-FS or LC-MS/MS has also been alternatively employed [104, 105].

In Uganda, Asiki et al. [60] reported human sera samples positive for AF-alb adducts in Southwestern Uganda. The AF-alb adduct ranged from 0 to 237.7 pg/mg alb among 100 adults (18–89 years) and 96 children (0–3 years) with 75% of the participants having AF-alb adduct levels above 7.1 pg/mg alb and 50% having levels above 10.3 pg/mg alb while 25% had levels above 15.1 pg/mg alb. Overall, all the adults and four children had detectable AF-alb adducts in the study. Respondents living close to trading centers had significantly (*p*=0.003) higher levels of detectable AF-alb adducts compared to their counterparts living in villages. Respondents consuming *matooke* (banana) had half detectable AF-albumin adduct compared to those who did not consume it. This is because these respondents are more likely to consume other foods which are prone to AF contamination; hence, people consuming *matooke* are less likely to have detectable AF-albumin adduct.

A longitudinal exposure study by Kang et al. [61] assessed AF exposure in Southwestern Uganda, reporting that 90% (642/713 of the sera) of samples drawn from the General Population Cohort were positive for AFB-Lys with a median level of 1.58 pg/mg and albumin range of 0.40–168 pg/mg. AFB-Lys adducts from 1999 to 2003 in the Rakai Community Cohort Study showed a detection rate of 92.5% (346/374) with a median of 1.18 pg/mg and a range of 0.40–122.5 pg/mg. Thus, it was deduced that AF exposure is high in the studied area and a similar finding is expected in other parts of Uganda. Further, a study done around the same time in the Northern part of Uganda [44] reported that there is a causal effect relationship between AF exposure and impaired growth in infants.

A cohort study by Lauer et al. [74] evaluated the association between maternal AF exposure during pregnancy and adverse birth outcomes, lower birth weight, in a sample of 220 mother-infant pairs in Mukono district, Uganda. Maternal AF exposure was assessed at 17.8 ± 3.5 pg/mg week gestation. Anthropometry and birth outcome characteristics were obtained within 48 hours of delivery. Median maternal AFB‐Lys level was 5.83 pg/mg alb (range: 0.71–95.60 pg/mg alb; interquartile range: 3.53–9.62 pg/mg alb). Increase in maternal AFB‐Lys levels was significantly associated with lower weight (*p*=0.040), lower weight‐for‐age *z*‐score (*p*=0.037), smaller head circumference (*p*=0.035), and lower head circumference‐for‐age *z*‐score (*p*=0.023) in infants at birth. The team concluded that there is a correlation between maternal AF exposure during pregnancy and adverse birth outcomes, particularly lower birth weight and smaller head circumference, though these warrant further studies.

### 3.3. Coexposure Assessment with Other Mycotoxins

The likelihood that mycotoxins may interact synergistically to induce amplified toxicity in animals is high because toxigenic fungi often occur simultaneously in the same batch of food/matrix and some fungi are capable of simultaneously producing several mycotoxins in a single given substrate. Unfortunately, there are no data in the open literature in Uganda reporting on the assessment of coexposure of AFs with other important mycotoxins such as fumonisins, ochratoxins, trichothecenes, and zearalenone. The paucity of this data is partially due to the underdevelopment of valid biomarkers [106]. Mycotoxin-specific biomarkers for common mycotoxins such as fumonisins and deoxynivalenol have been developed only very recently [107, 108], and their utilization in epidemiological studies can be termed as nascent. Therefore, there is a need for assessment of coexposure to aflatoxins in Uganda with other mycotoxins.

## 4. Prevention and Control

### 4.1. International, Regional, and Statutory Efforts

Efforts have been put on AF control in Uganda through countrywide awareness creation [109–111]. This is being done by the Eastern Africa Grain Council (EAGC) in collaboration with Uganda National Bureau of Standards (UNBS) through the Eastern Africa Grain Institute with its headquarters at Muyenga, Kampala. Between 2015 and 2018, maize exporters, traders, farmer-based organizations, and warehouse handlers were trained on understanding the integrated East African maize standard (EAS 2 : 2013), food standardization, comparison of East African standards with international standards, standard maize sampling methods, maize grading, mycotoxins, and the available methods for mycotoxin analysis [112].

Since its launch in 2006, EAGC has been leading the fight against AFs, working on a range of interventions to reduce the incidence, including assisting with the harmonization of AF control measures and improving the regulatory environment, running AF control training programs, providing moisture analyzers and tarpaulins to support farmers in drying and storing grains safely, sourcing for cheaper field-based AF testing kits and methods for measuring aflatoxins, conducting field surveys, regular analysis, and random sampling during harvesting at farm level to assess the prevalence and extent of contamination, working with East African Community to increase AF testing and surveillance in maize, participating in the development of the Partnership for Aflatoxin Control in Africa (PACA) strategy 2013–2022 as well as advising on the East African Community AF communication strategy [113].

National Agricultural Research Organization (NARO) in connection with Makerere University in 2010 developed a manual for the management of AF in peanuts [75]. The manual gives a general overview of AFs (structures, health, and economic effects), how to control AFs, and some of the farming practices in Uganda that favor AF growth. It was particularly drafted to provide ample guidance on the best practices in limiting AF contamination in peanuts and to raise the value of groundnuts and its products.

### 4.2. Scholarly Efforts

Probing investigations of Wacoo and his team [67] revealed that probiotic enrichment of a local maize-based traditional beverage (*kwete*) using starter culture with the probiotic *Lactobacillus rhamnosus* yoba 2012 and *Streptococcus thermophilus* C106 produced the beverage acceptable with consumers` acceptability score of greater/equal to 6 on a 9-point hedonic scale. The beverage remained stable for a month with reported *L*. *rhamnosus* counts of >10^8^ cfu/g, pH 3.9, and 0.6% w/v titratable acidity. AF analysis indicated that the water-soluble fraction of the beverage following fermentation had more than 1000-fold reduction in AFB_1_, AFB_2_, AFG_1_, and AFG_2_ initially spiked in the ingredients. The efficiency of *L. rhamnosus* to bind AFB_1_ was reported at 83.5% as determined by *in vitro* fluorescence spectroscopy.

Mold and total AF content of cereal flours and *obushera* (a local cereal-based beverage) from markets in metropolitan Kampala were evaluated by Byakika et al. [58]. The capacity of lactic acid bacteria (LAB) starters from *obushera*, *L. plantarum* MNC 21, *Weissella confusa* MNC 20, and *L. lactis* MNC 24 to bind AFB_1_ was evaluated against *L*. *rhamnosus* yoba 2012 (as a reference starter strain). The authors reported that mold counts in sorghum, millet, and *obushera* were between 0.0–2.4 log cfu/g, 2.0–6.5 log cfu/g, and 2.0–5.5 log cfu/g, respectively. The mold counts in all the flours as reported exceeded the maximum food safety compliance limit of 4.0 log cfu/g of molds; 88.0% of *obushera* had counts within the maximum compliance limit of 1.3 log cfu/g. Aflatoxigenic results revealed that total AF content of investigated matrices (sorghum, millet, and *obushera*), respectively, in *μ*g/kg were 22.3 ± 21.2, 9.9 ± 10.0, and 10.4 ± 6.1. The LAB bound 19.3–69.4% of AFB_1_ in a 1000 *μ*g/kg matrix, with binding efficiency in the order of *L. rhamnosus* yoba 2012 = *L. plantarum* MNC 21 > *W. confusa* MNC 20 = *L. lactis* MNC 24. The LAB-AFB_1_ complex was reportedly stable to physiological saline washes, indicating that the LAB with AF-binding properties can be harnessed for controlled fermentation to reduce AF content of *obushera* [58].

### 4.3. Suggested Management Strategies

The following control measures are suggested by this review for the control of AFs.

#### 4.3.1. Preharvest Management

Crop varieties that are less susceptible to fungal growth should be bred and planted. This has been reported to be one of the best approaches for reducing the effects of mycotoxin-producing fungal species [114]. Thus, local varieties of crops resistant to AF-producing fungi warrant investigation as some studies have unveiled that some local maize cultivars had lower AF levels than imported varieties [115]. In Uganda, Serenut 2 (a peanut variety) has been cited as a genetically more resistant variety to fungal growth and the production of AFs [76]. Drought, disease, and pest-tolerant/resistant crop varieties have been found to greatly reduce AF contamination. More so, host and parasite macro- and micromolecular trafficking that suggests the possibility to circumvent the AF problem by use of cross-species RNA interference has been suggested. This equips particularly maize with molecules that shuts down AF biosynthesis upon infection with aflatoxigenic fungi, thwarting AF accumulation.

Timely harvesting of grains with the husks upon maturity in dry conditions and early removal of any damaged maize kernels or cobs is a feasible AF reduction strategy [115].

Visual sorting, winnowing, washing, crushing, and dehulling have been found to contribute up to a 40–80% reduction in AF levels in grains [116, 117]. Sorting is highly recommended for reducing AF content in foods, peculiarly in peanuts [115, 118–120] and cassava. However, sorting and giving children the molded peanuts (called “*lake*” in Northern Uganda) or using them for making peanut paste should be discouraged. Sorting can be done using clean water; the damaged seeds or grains are buoyant while good ones sink and can be cooked directly. This is traditionally practiced in Northern Uganda with beans, peas, and cowpeas. Soaking and cooking in magadi soda, malting, and roasting are other methods that have been used to reduce the levels of AFs in maize [117, 121–123]. Magadi soda is unknowingly used by the rural community of Lango subregion as a catalyst for fastening the cooking of beans, peas, white ants, and sesame-based dishes (*alakena* and *agwaca*), vegetables, and sometimes cassava.

Protection of crops from pest attack is key in aflatoxin management. This can be done using ash while in storage as is done in maize [124, 125] and plant essential oils such as *Eucalyptus saligna* that have reported bioinsecticidal activity [126].

Biocontrol strategies employing concoctions from plants have been investigated and reported to inhibit *A. flavus* mycelial growth and proliferation. Essential oils of *Azadirachta indica* (neem) and *Morinda lucida* have been reported to retard aflatoxigenic *A. flavus* growth and its AF biosynthesis potential in inoculated maize grains [127]. Powder of *Aframomum danielli* (Zingiberaceae) can regulate molds and insect infestation in maize and soybeans in storage for over a year under ambient conditions [128].

Competitive exclusion has been reported as a feasible AF control strategy. *A. flavus* strains differ in AF production and this influences their crop contamination potential. The toxigenic strains (“S” strains) produce a lot of AFs with numerous small sclerotia (<400 *μ*m) whereas the “L” strains are atoxigenic and comparatively produce lower AF levels and a few large sclerotia that are >400 *μ*m [129]. There is always competitive exclusion when one strain competes to exclude another in the environment. Thus, a shift of strain profile from toxigenic to atoxigenic is a viable biological control strategy. This competitive exclusion strategy has yielded good results in some investigations with up to 96% reduction in AF levels [129].

A biopesticide, consisting of a rhizosphere-competent nonaflatoxigenic strain of *Aspergillus* with competitive saprophytic ability, may competitively exclude toxigenic strains from infecting the crop. Fluorescent pseudomonads and several strains of *Trichoderma* species inhabit the rhizosphere of many crop plants and have been identified as potentially promising biocontrol agents against *A. flavus.* Since the beginning of the 21^st^ century, many *Trichoderma* (>250) and *Pseudomonas* (>100) species have been isolated from peanut rhizosphere and evaluated for their antagonism towards *A. flavus* and their ability to reduce preharvest kernel infection of peanuts. Significant reduction of *A. flavus* populations and kernel infection occurred in both greenhouse and field experiments. Two *Trichoderma* isolates, Tv 47 and Tv 23, and two bacterial isolates, *P. cepacia* (B 33) and *P. fluorescens* (Pf 2), were effective in reducing aflatoxin content in the kernels. Control of AF contamination has also been reported to be effective using nonaflatoxigenic biocontrol *A. flavus* strains that outcompete the wild strain, reducing their concentration at the contaminated site [130]. However, the efficacy of these agents warrants establishment under Ugandan conditions so that affordable, readily available, and effective formulations can be developed for use.

#### 4.3.2. Postharvest Management

The cost of prevention versus the cost of cure is not a new debate, and thus, some cure technologies for AFs are in place. One of the credited strategies is to reduce the moisture content of harvested food crops to safe storage levels (12–14%). Harvested crops should be shelled and cleaned prior to storage to reduce incidences of pest infestation which may promote aflatoxigenic contamination [59]. Further, storage facilities should be well ventilated to ensure temperatures between 25°C and 32°C and sustained relative humidity above 65% suitable for aflatoxin growth are not attained [131]. According to Sumner and Lee [132], temperatures below 18°C and moisture of 12–13% usually stop the development of *Aspergillus* fungi.

Clays such as Novasil Plus (NSP; BASF Corp., Ludwigshafen, Germany) have been demonstrated to bind AF in animal feeds [133] and reduce its content. An innovation for postharvest AF elimination called the “Toxin Scrub” has been demonstrated by Grain and Toxins Ltd in Uganda, but its usage has been delimited by its prohibitive cost [47]. The technology utilizes ozone, a strong oxidizer to eliminate nearly all the mycotoxins in the grain. This is supported by the fact that AFs are unstable to UV light in the presence of oxygen, to extremes of pH (<3, >10) and to oxidizing agents such as sodium hypochlorite, potassium permanganate, chlorine, hydrogen peroxide, ozone, and sodium perborate [20]. AFs are also degraded by reaction with ammonia, various amines, and sodium hypochlorite. Some compounds such as curcumin can alter the microsomal activation of AFB_1_ and reduce the AFB_1_ toxicity by increasing its detoxification.

Chemoprotection against AFs consumed by animals has also been reported. It utilizes compounds such as esterified glucomanoses and other yeast extracts that increase the animal's detoxification process or otherwise prevent the production of AF-epoxide, thereby reducing or blocking AFB_1_-induced hepatocarcinogenesis. Oltipraz and chlorophyll are used to reduce the biologically effective dose and act by binding AFs, thereby rendering them biologically unavailable to humans and animals.

### 4.4. Treatment of Aflatoxicosis

No scientifically proven specific antidote for ingested AFs has been reported. However, the timely use of l-methionine (200 mg/kg) and sodium thiosulfate (50 mg/kg) after every 8 hours has reported therapeutic significance. Dietary intake of protein, vitamins, and antioxidants can be encouraged in case of aflatoxicosis [134].

## 5. Conclusion

Aflatoxin surveillance in Uganda is done through a reactive approach. Ugandan foods are mycotoxicologically contaminated with aflatoxins, and this has serious health implications. No study in Uganda has assessed AFs in beers, imported rice such as basmati, and sugarcane despite them being daily consumables. The Ugandan government through its ministries should develop the capacity to detect, quantify, monitor, and regulate AFs in foods produced and sold within the country and those exported/imported. There is a need for more aflatoxin exposure assessments as well as coexposure to aflatoxins with other mycotoxins.

## Figures and Tables

**Table 1:** Types and chemical structure of common aflatoxins.

Difuranocoumarins	Aflatoxin	Chemical structure and molecular formula	Molecular weight (kg/mol)	Metabolites
Difurocoumarocyclopentenone series	AFB_1_	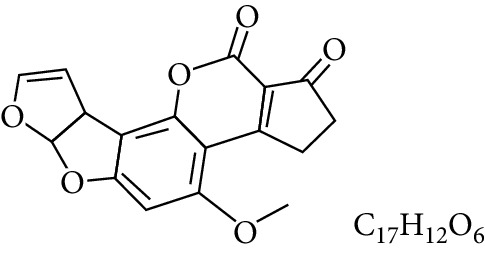	312.274	
	AFB_2_	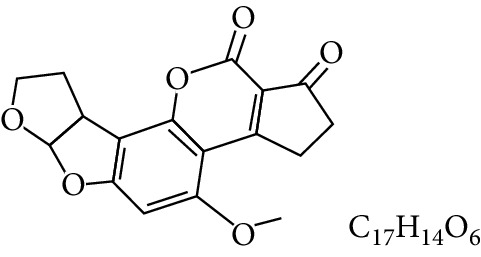	314.2895	
	AFB_2A_	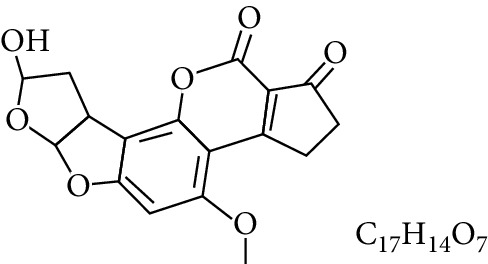	330.2889	
	AFM_1_	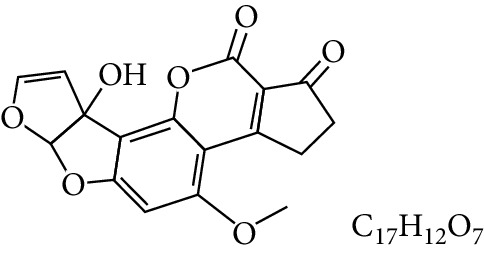	328.273	Metabolite of AFB_1_ in humans and animals comes from the mother's milk. It is believed to be associated with the casein fraction of milk
	AFM_2_	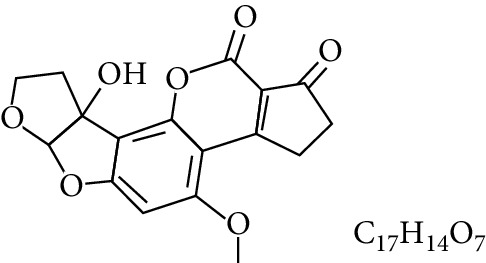	330.2889	Metabolite of aflatoxin B_2_ in milk of cattle fed on AF-contaminated foods
	AFM_2A_	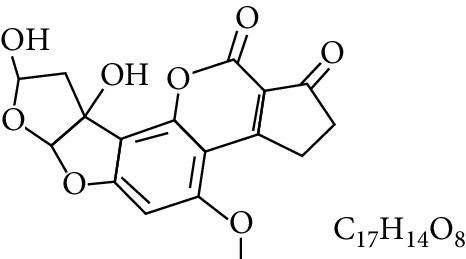	346.069	Metabolite of AFM_2_
	Aflatoxicol (AFL)	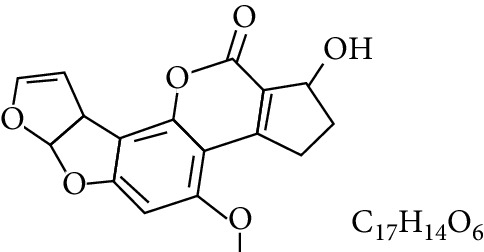	314.289	Metabolite of AFB_1_
	Aflatoxicol M_1_	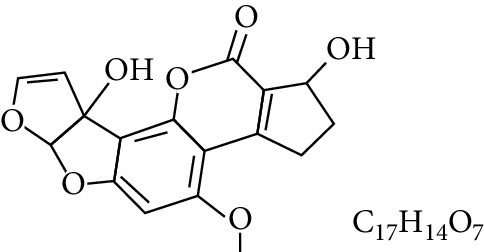	330.2889	Metabolite of AFM_1_
Difurocoumarolactone series	AFG_1_	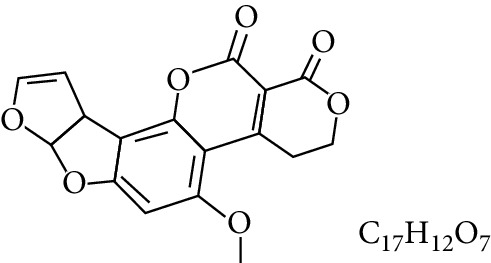	328.273	
	AFG_2_	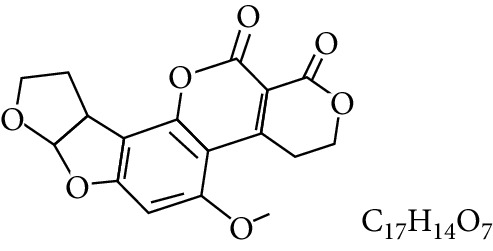	330.289	
	AFG_2A_	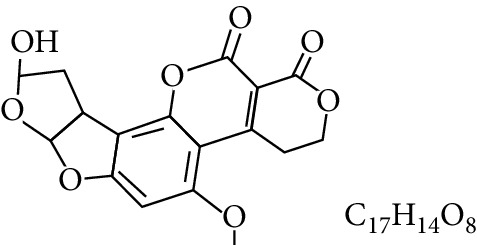	346.2883	Metabolite of AFG_2_
	AFGM_1_	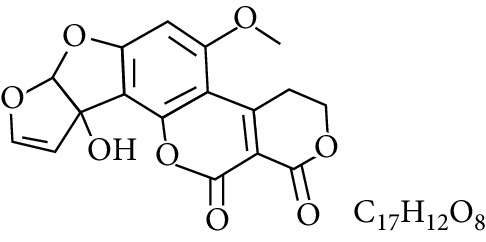	344.272	
	AFGM_2_	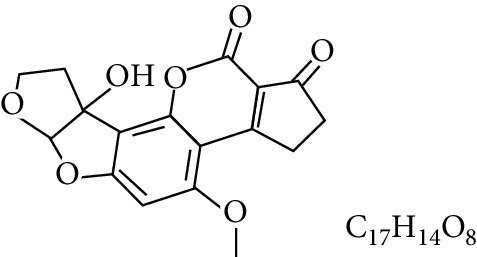	330.2889	Metabolite of AFG_2_
	AFB_3_ (parasiticol)	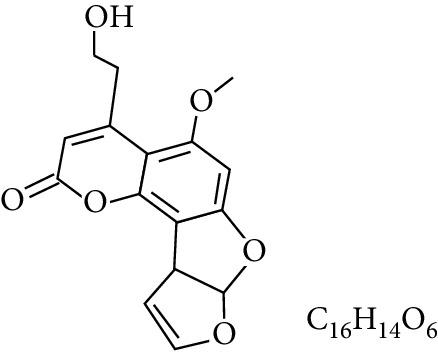	302.279	
	Aflatrem	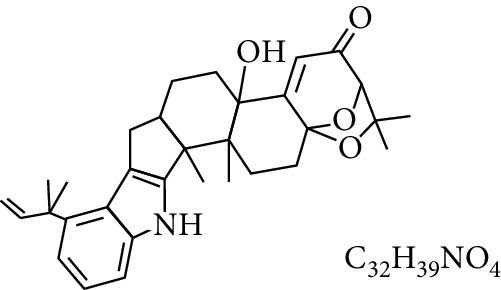	501.656	
	Aspertoxin	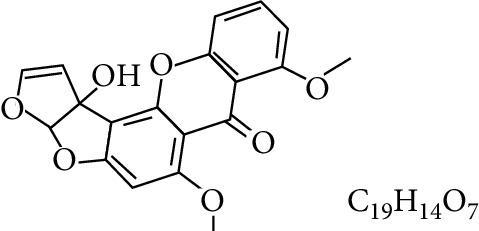	354.310	
	AFQ_1_	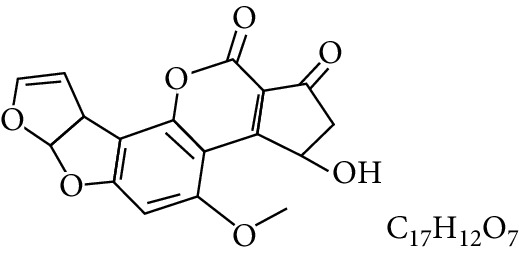	328.273	Major metabolite of AFB_1_ in *in vitro* liver preparations of other higher vertebrates

Source: modified after [9, 10].

**Table 2:** Median lethal dose for AFB_1_ administered as a single dose to different animals.

Animal	Sex	Age/size	LD_50_ (mg/kg body weight)
Golden hamster	Male	30 days	10.2
Rat	Male/female	1 day	1.0
Rat	Male	21 days	5.5
Rat	Female	21 days	7.4
Rat	Male	0.001 kg	17.5
Dog	Male/female	Adult	0.5
Pig	Unspecified	6-7 kg	0.6
Chicken embryo	Unknown	Not applicable	0.025
Duckling	Male	1 day	0.37

Source: Agag [38], Ciegler [10], and Robens and Richard [39]. 1 mg/kg = 1000 *μ*g/kg.

**Table 3:** Aflatoxin content of peanuts from farmers in some selected peanut growing districts of Uganda.

Village (district)	Samples analyzed	Aflatoxin status		Aflatoxin concentration (*μ*g/kg)
		Positive (%)	Negative (%)	
Kabulamuliro (Mubende)	*n* = 25	80	20	12.4 ± 5.31
Kiboyo (Iganga)	*n* = 20	75	15	10.5 ± 6.15
Bugodi (Mayuge)	*n* = 15	60	40	7.3 ± 4.98
Gayaza (Mubende)	*n* = 12	67	33	9.8 ± 4.32

Adapted from Kaaya et al. [79].

**Table 4:** Total AF content of some selected foods in some Ankole districts of Southwestern Uganda.

Matrix/food sample	Samples analyzed	% AF-positive samples		Average total AF (*μ*g/kg)
		<4.0 *μ*g/kg (%)	>4.0 *μ*g/kg (%)	
Peanut flour	*n* = 3	0	100	11.5 ± 0.43
Sorghum (flour and porridge)	*n* = 7 and *n* = 15	42. 9 and 13.3	57.1 and 86.7	15.2 ± 0.20
Millet (flour and porridge)	*n* = 12 and *n* = 21	25 and 0	75 and 100	14.0 ± 1.22
Cassava flour	*n* = 18	38.9	61.1	16.0 ± 1.66
*Eshabwe* (porridge) sauce	*n* = 14	7.1	92.9	18.6 ± 2.40

Excerpted from Kitya et al. [57].

**Table 5:** Per capita food and aflatoxin contamination patterns in the East African region.

Food	Country	Per capita food consumption (g/person/day)	Mean AF content (*μ*g/kg)
Maize	Uganda	400	9.7
	Tanzania	69	49.7
	Kenya	405	131.7
Groundnuts (peanuts)	Uganda		25.1
	Tanzania		15.0
	Burundi	65	12.5
Cassava chips	Uganda		0.5
	Tanzania	214	0.9
Sorghum	Tanzania	40	3.0
Milk	Kenya	750 ml	0.8
	Tanzania	750 ml	0.9

Adapted from the report by the East African Community's aflatoxin working group in April 2013 (Dar es Salaam-Tanzania, EAC/TF/405/2013) cited in a penultimate study [82].

**Table 6:** Aflatoxin content of some staple foods in Uganda.

Sample/matrix	Number of samples			Total aflatoxin (*μ*g/kg)		
	Analyzed	AF positive	% AF positive	1–100	100–1000	>1000
Beans	64	46	71.9	30	11	5
Maize	49	22	44.9	13	9	0
Sorghum	69	26	37.7	19	5	5
Peanuts	152	27	17.8	11	8	8
Millet	55	9	16.4	9	0	0
Peas	19	3	15.8	3	0	0
Cassava	34	4	11.8	0	2	2
Rice	11	0	N/A	0	0	0
Other grains	11	2	18.2	0	1	1
Grain mixtures	16	3	18.7	2	0	0
Total	480	142		87	37	18

Adapted from [93]. N/A: not applicable.

**Table 7:** Hepatoma incidence and frequency of aflatoxin contamination of some staple foods in Uganda.

Region^a^	Hepatoma cases/100,000 people per annum	Aflatoxigenic contamination				
		Analyzed samples	% of AF-positive samples	Total aflatoxin (*μ*g/kg)		
				1–100	100–1000	>1000
Toro	No data collected	29	79.3	10	31	38
Karamoja	15.0	105	43.8	24	15	5
Buganda	2.0–3.0	149	28.9	23	4	1
West Nile	2.7	26	23.1	19	4	0
Busoga	2.4	39	10.3	05	5	0
Acholi	2.7	26	15.4	15	0	0
Ankole	1.4	37	10.8	11	0	0
Rwanda immigrants	3.0	None collected	Not applicable	Not applicable	Not applicable	Not applicable

Modified from [93]. Regions have different tribes with different traditional practices and ways of handling foods. ^a^Uganda is no longer divided into these regions, which have instead been made districts.

**Table 8:** Some of the analytical methods employed by aflatoxigenic investigations in Uganda.

Method	Sample (s)	Year^a^	References
Lateral flow immunochromatography	Maize grain	2019	[62]
HPLC	Maize-based product (*Kwete*)	2019	[67]
ELISA	Sorghum, millet, *obushera*	2019	[58]
ELISA, HPLC	Maize flour	2018	[64]
ELISA	Maize, sorghum, millet, sesame, peanuts	2018	[63]
HPLC	Human sera	2018	[92]
TLC, ELISA	Peanuts (seeds and paste)	2017	[65]
LC/MS/MS	Peanuts (seeds and paste)	2017	[66]
FS	Peanuts (seeds and paste), cassava flour, maize grains	2016	[78]
ELISA	Human sera	2015	[61]
ELISA	Human sera	2014	[60]
ELISA	Cereal-based baby foods	2011	[95]
FS	Cassava	2010	[71]
FS	Sorghum, millet, *Eshabwe,* peanut (seeds and paste), cassava chips	2010	[57]
ELISA	Maize	2006	[96]
FS	Peanuts	2006	[79]

^a^Years cited represent the years the data were published with most data collected in over 2 months to 1 year.
